# The Dyadic Effects of Perceived Support on Depression in Spousal Care Partners

**DOI:** 10.1093/geroni/igaa057.1139

**Published:** 2020-12-16

**Authors:** Kylie Meyer, Neela Patel, Carole White

**Affiliations:** 1 UT Health Sciences at San Antonio, San Antonio, Texas, United States; 2 UT Health San Antonio, San Antonio, Texas, United States

## Abstract

Relationship quality is an important factor affecting care partners’ health and wellbeing. Supportive marital relationships are associated with better physical and subjective health, whereas strain is associated with poorer health. Recent studies now indicate a dyadic effect of relationship quality on health outcomes, such that an individual’s perceptions of their relationship also affects their partner’s outcomes. Few studies have examined the dyadic effects of relationship quality on mental health among older cognitively intact caregiving couples. To address the lack of dyadic research about how perceived support from one’s spouse related to experiences of depression for individuals and their care partners, we apply cross-sectional actor partner interdependence models (APIMs) to data from the Health and Retirement Study (HRS) (N=490 dyads). APIM regression models controlled for participant demographic characteristics, relationship length, and care recipient functional ability. Findings showed that positive perceived support from a spouse had a stronger negative association with one’s own depression for care recipients than for caregivers. Similarly, greater negative perceived support from a partner was associated with higher levels of depression; whether the partner was the caregiver or care recipient did not make a difference in this model. Although there are hundreds of caregiver interventions to address caregivers’ mental health, few have demonstrated improvement in care recipient outcomes. Observation of both actor and partner effects in this study suggests there may be opportunities to improve care recipient and caregiver mental health by targeting interventions to promote high quality relationships with caregivers or both members of the care dyad.

